# Posttranslational modifications of blood-derived alpha-synuclein as biochemical markers for Parkinson’s disease

**DOI:** 10.1038/s41598-017-14175-5

**Published:** 2017-10-20

**Authors:** Hugo Vicente Miranda, Rafaela Cássio, Leonor Correia-Guedes, Marcos António Gomes, Ana Chegão, Elisa Miranda, Tiago Soares, Miguel Coelho, Mário Miguel Rosa, Joaquim J. Ferreira, Tiago Fleming Outeiro

**Affiliations:** 1 CEDOC, Chronic Diseases Research Centre, NOVA Medical School|Faculdade de Ciências Médicas, Universidade NOVA de Lisboa, Campo dos Mártires da Pátria, 130, 1169-056 Lisboa, Portugal; 2 Instituto de Medicina Molecular, Faculdade de Medicina, Universidade de Lisboa, Lisboa, Portugal; 3 Clinical Pharmacology Unit, Instituto de Medicina Molecular, Lisbon, Portugal; 4 Department of Neurosciences and Mental Health, Neurology, Hospital de Santa Maria-CHLN, Lisbon, Portugal; 5 Department of Experimental Neurodegeneration, Center for Nanoscale Microscopy and Molecular Physiology of the Brain (CNMPB), Center for Biostructural Imaging of Neurodegeneration, University Medical Center Göttingen, Waldweg 33, 37073 Göttingen, Germany; 6 Max Planck Institute for Experimental Medicine, Göttingen, Germany

## Abstract

Parkinson’s disease (PD) is a progressive neurodegenerative disorder known for the typical motor features associated. Pathologically, it is characterized by the intracellular accumulation of alpha-synuclein (aSyn) in Lewy bodies and Lewy neurites. Currently, there are no established biochemical markers for diagnosing or for following disease progression, a major limitation for the clinical practice. Posttranslational modifications (PTMs) in aSyn have been identified and implicated on its pathobiology. Since aSyn is abundant in blood erythrocytes, we aimed to evaluate whether PTMs of aSyn in the blood might hold value as a biomarker for PD. We examined 58 patients with PD and 30 healthy age-matched individuals. We found that the levels of Y125 phosphorylated, Y39 nitrated, and glycated aSyn were increased in PD, while those of SUMO were reduced. A combinatory analysis of the levels of these PTMs resulted in an increased sensitivity, with an area under curve (AUC) of 0.843 for PD versus healthy controls, and correlated with disease severity and duration. We conclude that the levels of these selected PTMs hold strong potential as biochemical markers for PD. Ultimately, our findings might facilitate the monitoring of disease progression in clinical trials, opening the possibility for developing more effective therapies against PD.

## Introduction

Parkinson’s disease (PD) is the second most common progressive neurodegenerative disorder affecting 1–2% of people over the age of 65 years^1^. The diagnosis of PD is currently based on typical clinical motor features, such as bradykinesia, resting tremor, or rigidity^2^. The response to levodopa treatment represents an important factor for diagnosis, since PD patients usually present a good response to this medication^3^. Different clinical scales are routinely used to classify and stage the disease. The Movement Disorder Society (MDS) recently revised the Unified PD Rating Scale (MDS-UPDRS), which is subdivided into four components^4^. Parts I and II are associated to the non-motor and motor experiences of everyday, while part III is reserved to the motor evaluation and finally Part IV is related to motor complications^5^. The Hoehn and Yahr (HY) scale is divided into 5 stages that reflect motor progression of the disease^6^. However, misdiagnosis of PD is rather common^7,8^, affecting therapeutics and complicating patient selection for clinical trials. Thus, it is urgent to identify novel and objective biomarkers to unequivocally diagnose and follow disease progression.

Pathologically, PD is characterized by the loss of nigrostriatal dopaminergic neurons and the accumulation of neuronal cytoplasmic inclusions known as Lewy bodies (LBs) or Lewy neurites, which are primarily composed of the protein alpha-synuclein (aSyn)^9^. The majority of PD cases are sporadic, however several genes have been associated with familial cases^10^. Mutations and multiplications in the gene encoding for aSyn are also associated with familial forms of PD^11^. Interestingly, in both sporadic and familial forms of PD the progression and severity of the disease correlate with the distribution of aSyn inclusions^12^.

For reasons we still do not fully understand, aSyn is prone to misfolding and self-association into high molecular weight species, ultimately forming LBs^13^. Posttranslational modifications (PTMs) have a direct impact in the aggregation and toxicity of aSyn. The protein undergoes several PTMs such as acetylation and glycation, as we recently described^14,15^, as well as oxidation, phosphorylation, nitration, sumoylation, and ubiquitination^16–18^. We showed that while acetylation protects from aSyn aggregation and toxicity^14^, glycation exacerbates oligomerization and cytotoxicity, inducing dopaminergic neuronal death^15^. Phosphorylation has been extensively studied PTM since most aSyn found in LBs is phosphorylated on serine 129^19,20^. However, the effects of S129 phosphorylation are still controversial^21–25^. Other modifications, such as nitration^26^ and oxidation^27^ enhance, while sumoylation^28^ prevents/reduces the aggregation of aSyn. Nevertheless, although PTMs may play important roles on aSyn biology, our understanding of the precise effects of PTMs on aSyn is still limited.

aSyn is present in various body fluids such as cerebrospinal fluid (CSF) and blood^29,30^. Thus, the presence of this protein in easily-accessible body fluids opens the possibility of investigating PTMs in aSyn. Taking advantage of the thermo-stability of aSyn^31^, we detected PTMs in aSyn extracted from brain tissue or from cultured cells^14,15,32^.

Although the pool of aSyn in the blood may differ from that in the brain or CSF, we hypothesized that, given the central role this protein plays in PD, it might report on disease-related alterations that may be used as biomarkers for PD. This is in line with the observation that aSyn can be secreted from neuronal cells^33^, and that it is phosphorylated on serine 129 in the serum of PD patients^34^.

In this study, we identified a specific pattern of aSyn PTMs that discriminated PD patients from controls individuals. As these PTMs correlate with disease severity and duration, we propose that these modifications may possibly be used as biochemical markers for PD.

## Results

### Patient population and demographics

We recruited a group of 88 individuals, divided into 3 subgroups: 28 diagnosed with PD for 2–4 years, 30 diagnosed for ≥10 years, and 30 controls. Blood samples were processed by the same personnel. The PD group diagnosed with the disease for 2–4 years presented a male to female (M:F) ratio of 1: 1, a mean age at onset of 69.0 ± 10.3 years, a mean disease duration of 3.1 ± 0.7 years, a MDS-UPDRS III of 38.4 ± 10.8 and a HY of 1.6 ± 0.8. The PD group diagnosed for ≥10 years presented a M:F ratio of 1: 1, a mean age of onset of 67.2 ± 7.6 years, a mean disease duration of 17.7 ± 4.3, a MDS-UPDRS III of 60.0 ± 19.9 and a HY of 3.0 ± 1.1. The mean age of controls was 67.2 ± 7.6 years. Male to female ratio in the control group was 1:2 (Table 1 and Fig. S1).

**Table 1:** Demographics data of recruited individuals.

	Whole Cohort		Subset with PD	
	Con	PD	2–4 years	≥ 10 years
Subject number	30	58	28	30
Age (range)	67.4 ± 9.0 (52–83)	68.1 ± 9.0 (48–84)	69.0 ± 10.3 (48-84)	67.2 ± 7.6 (53–80)
Gender (male: female)	0.5 (10: 20)	1.0 (29: 29)	1.0 (14: 14)	1.0 (15: 15)
MDS-UPDRS III (range)		49.6 ± 19.4 (10–97)	38.4 ± 11.0 (10–58)	60.0 ± 19.9 (28–97)
HY (range)		2.4 ± 1.2 (0–5)	1.6 ± 0.8 (0–4)	3.0 ± 1.1 (2–5)
Disease Duration, year (range)		10.8 ± 7.9 (2–27)	3.1 ± 0.7 (2–4)	17.7 ± 4.3 (10–27)

*Con* control, *PD* Parkinson’s disease, MDS-*UPDRS III* Movement Disorders Society Unified Parkinson’s disease Rating Scale part III, *HY* Hoehn and Yahr.

### aSyn purification and enrichment

**Figure 1:**
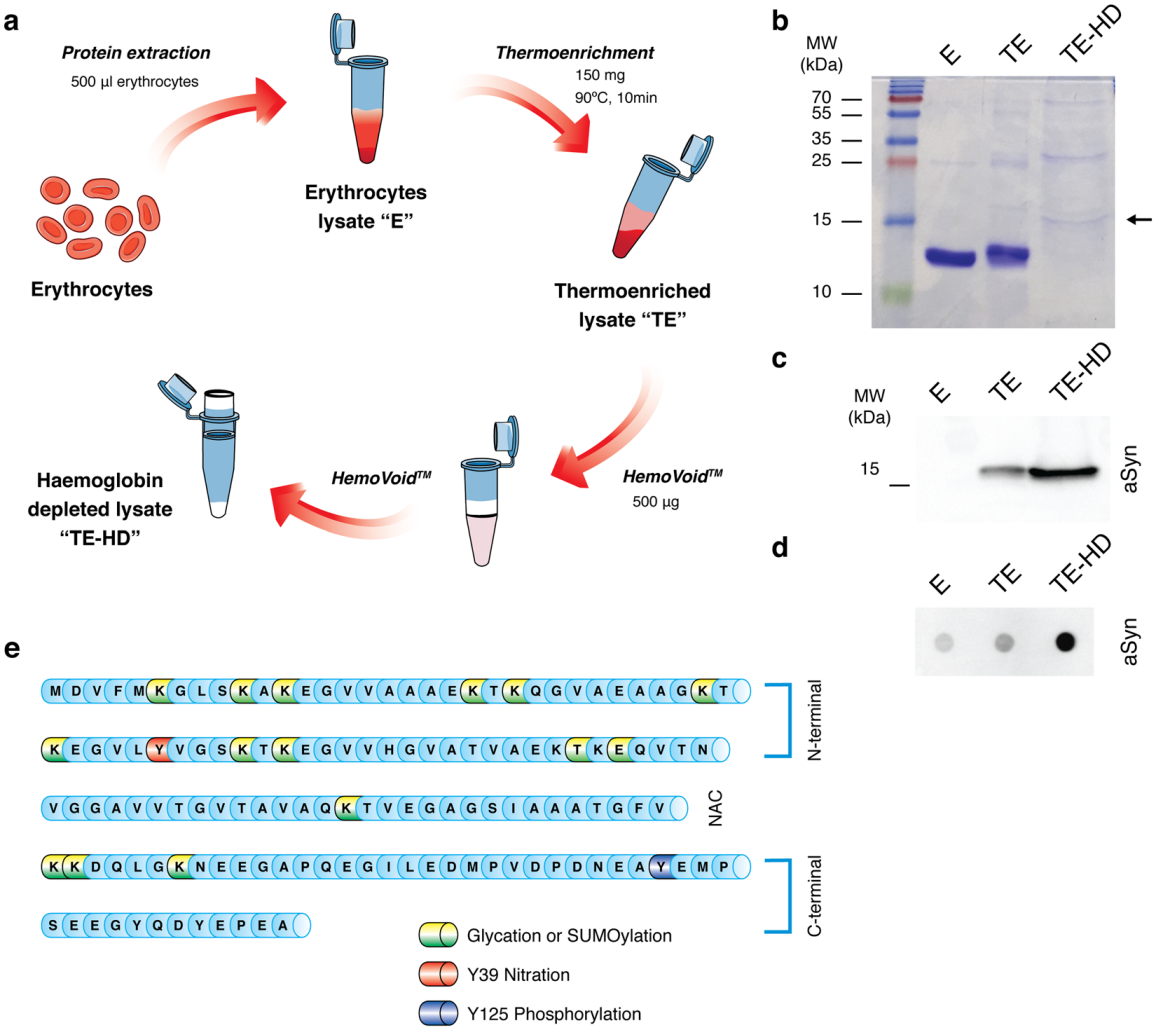
Partial purification and enrichment of aSyn from erythrocyte lysates for PTM analyses. (**a**) Schematic of aSyn enrichment. Erythrocyte crude lysates (E) are first thermo-enriched (TE) and then haemoglobin-depleted (TE-HD). (**b**) Coomassie staining of an SDS-PAGE separation of 5 μg of E, TE and TE-HD. Arrow indicates the MW of aSyn. (**c**) Western blot analysis of 5 μg of E, TE and TE-H probed with an antibody against aSyn. (**d**) Dot blot analysis of 5 μg of E, TE and TE-HD extracts probed with an antibody against aSyn. (**e**) Schematic of the aSyn protein sequence highlighting the PTMs analysed: Glycation (yellow), SUMOylation (green), nY39 (red) and pY125 (blue).

To analyse PTMs of aSyn in blood, we first enriched the aSyn content from erythrocyte lysates taking advantage of the thermo-stability of aSyn, as previously described^32^. Briefly, as described in Fig. 1a–d, by heating protein samples, non-thermo-stable proteins precipitated, while aSyn remained in the soluble fraction, enabling its detection (Fig. 1b–d). As haemoglobin is the major protein component of erythrocytes lysates (90%), we depleted this protein from thermo-enriched erythrocyte lysates using HemoVoid, a silica-based protein enrichment matrix that removes haemoglobin from lysates, enabling the additional concentration of other proteins of lower abundance^35^. We confirmed the aSyn enrichment by immunoblotting (SDS-PAGE and dot-blot) (Fig. 1c,d). Thus, for all subsequent analysis, the erythrocyte fractions were thermo-enriched and haemoglobin depleted. Although the amount of haemoglobin is severely reduced in thermo-enriched-haemoglobin-depleted (TE-HD) extracts, and a band at 15 kDa is evident, other higher molecular weight bands are also observed (Fig. 1b).

### aSyn in the blood is posttranslationally modified

**Figure 2:**
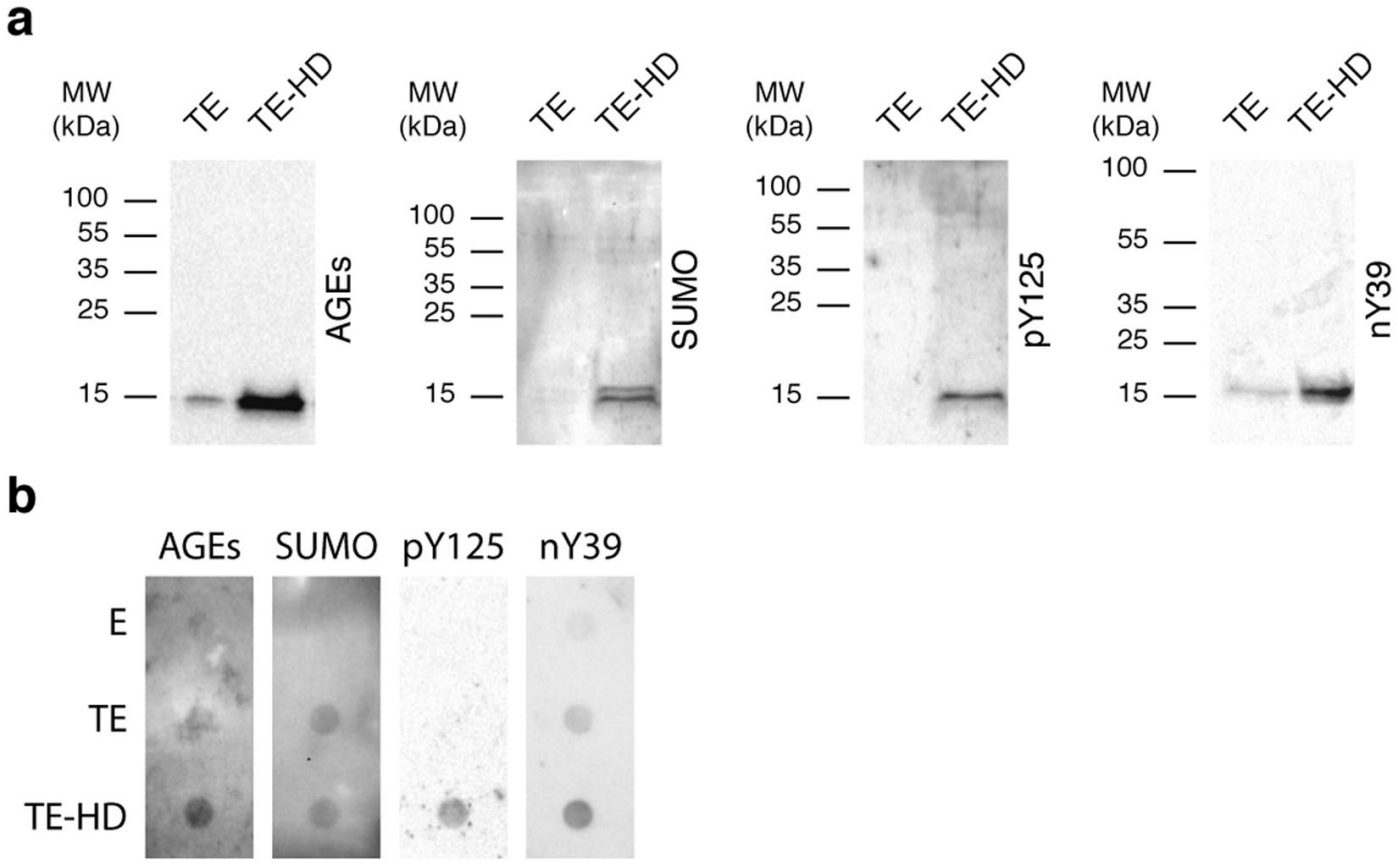
Detection of PTMs in the thermo-enriched-haemoglobin-depleted extracts. (**a**) Western blot analysis of an SDS-PAGE separation of 5 μg of TE and TE-HD probed with antibodies against AGEs, SUMO-1, pY125 or nY39. (**b**) Immunoblot analysis of 5 μg of E, TE and TE-HD applied in a dot blot probed as in (**a**).

First, we selected a set of candidate PTMs that could be assessed by immunoblotting analyses - tyrosine (Y) 125 phosphorylation (pY125), Y39 nitration (nY39), lysine (K) glycation (AGE), and SUMOylation (SUMO-1) (Fig. 1e). We analysed the PTMs in thermo-enriched (TE) and in TE-HD extracts and found only weak signal for all PTMs in TE samples (Fig. 2a,b). In contrast, the signal for all PTMs was much stronger in TE-HD (Fig. 2a,b). Moreover, for the case of pY125, nY39 and AGE, the signal occurred at the molecular weight corresponding to aSyn. This suggests that, in the TE-HD extracts, the majority of the signal for the PTMs should correspond to modified aSyn, and not to other unspecific proteins, since most proteins are eliminated in the TE step. In the case of SUMO-1, we only detected signal at ~15 kDa, which is lower than the expected molecular weight for sumoylated aSyn (~27 kDa). As a control, we also analysed ubiquitination in the same samples (Fig. S2). While we detected both free ubiquitin and ubiquitinated proteins in the TE extracts, no ubiquitin is detected in the TE-HD extracts. This indicates that only specific modifications are detected in the TE-HD. Importantly, in crude erythrocyte extracts (E), almost no signal was detected for the PTMs tested. Since aSyn was clearly enriched in this fraction, we hypothesized that the PTMs, but SUMO-1, are likely aSyn specific.

**Figure 3:**
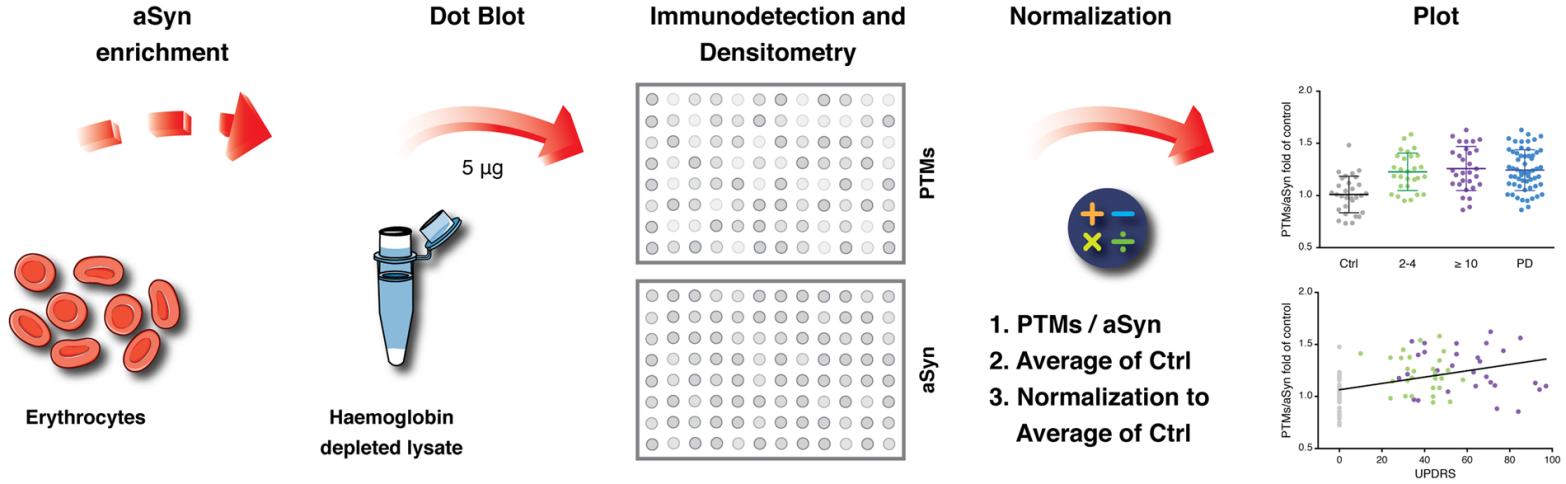
Schematic of the measurement of the levels of the PTMs in erythrocytes. After the enrichment procedure described in Fig. 1a, TE-HD extracts are applied onto a nitrocellulose membrane using a dot blot system. The membranes are probed for the PTMs of interest using specific antibodies and, after stripping, for total aSyn. The ratio between the PTMs and the levels of aSyn is determined. The average corresponding to the control group is then used to normalize all levels. Data is presented as PTM/aSyn fold of control.

**Figure 4:**
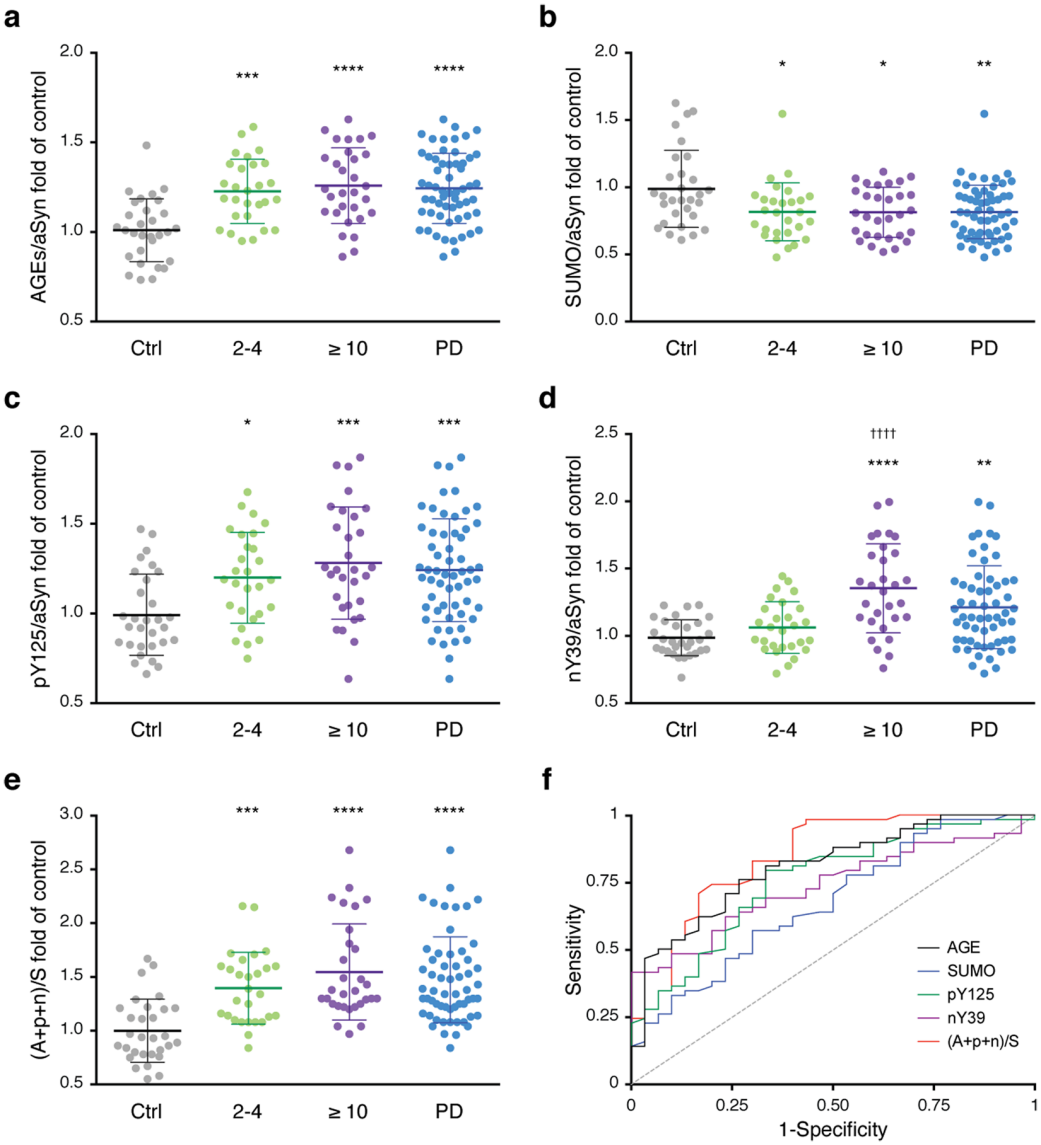
The levels of the PTMs are specifically altered in PD patients. Individual mean values of glycation (**a**), SUMOylation (**b**), pY125 (**c**), nY39 (**d**) and combinatory analysis of PTMs (**e**) of the healthy individuals (Ctrl, grey) or the different PD groups (2–4, green; ≥ 10 years, purple; or all PD patients, blue) are presented. Data was normalized to the levels of aSyn of each corresponding individual, and to the mean level of the Ctrl group (at least n = 4). *p < 0.05, **p < 0.01, ***p < 0.001, ****p < 0.0001 (comparison with Ctrl); ^††††^ *p* < 0.0001 (comparison with 2-4 years). (**f**) ROC curve to evaluate the utility of the PTMs in discriminating patients with PD from healthy controls. Glycation (black), SUMO-1 (blue), pY125 (green), nY39 (purple) and PTMs combination (red) curves are presented.

To assess the levels of these PTMs, we used the methodology described in Fig. 3. Briefly, we applied the enriched protein lysates in a dot-blot system onto nitrocellulose membranes. Afterwards, we probed for these modifications and for the total levels of aSyn by immunoblotting. The levels of each PTM were normalized to the corresponding levels of aSyn from each individual. Importantly, we could not verify significant differences in the total levels of aSyn between individuals (Fig. S3). Interestingly, we detected significant changes in the levels of these PTMs in PD patients. In the case of advanced glycation end-products (AGEs), the levels were increased in the PD group (1.24 fold) (Fig. 4a). Both the early-diagnosed patients (2–4 years) and the patients with established disease (≥10 years) displayed a significant increase in the levels of AGEs (1.22 and 1.25-fold, respectively) (Fig. 4a). The levels of SUMO-1 were decreased in both groups of patients (0.83 in 2–4 years; 0.82 in ≥10 years; 0.83 in PD patients) (Fig. 4b). The levels of pY125 were increased (1.20 in 2–4 years; 1.29 in ≥10 years; 1.25 in PD patients) (Fig. 4c). Finally, the levels of nY39 were also increased in PD patients (1.22 fold), and were even higher in the group diagnosed for ≥10 years (1.36 fold) (Fig. 4d).

To integrate all data, we also analysed the results as the ratio between the sum of the PTMs that increased and the PTM that decreased [(AGEs + pY125 + nY39) / SUMO]). Strikingly, the separation between control individuals and PD patients was even more evident (1.47 fold), as was the separation between the two subgroups of PD (1.40 in 2–4 years; 1.55 in ≥ 10 years) (Fig. 4e).

Importantly, the individual levels of AGEs, SUMO-1, pY125, nY39 or the combined levels of these PTMs [(AGEs + pY125 + nY39) / SUMO] showed no association with sampling age (Fig. S4) or gender (Fig. S5).

To further evaluate the utility of measuring these PTMs as a mean of discriminating between patients with PD and healthy controls, we performed a receiver operating curve (ROC) curve analysis. The area under the curve (AUC) provides a predictive value of the diagnostic potential of the measurements. A maximum AUC = 1 indicates a perfect diagnostic differentiation between diseased and control individuals, while an AUC = 0.5 indicates a test with no discrimination^36^. Notably, all PTMs presented an AUC of ≥0.7, indicative of a strong diagnostic potential. The best-performing PTM is glycation (measured by the levels of AGEs) (AUC = 0.807 ± 0.0479, 95% confidence interval 0.713–0.900, *p* < 0.0001). The AUC of the combined PTMs [(AGEs + pY125 + nY39) / SUMO] resulted in an AUC of 0.843 ± 0.0461 (95% confidence interval 0.743–0.934, *p* < 0.0001) indicative of high discriminatory potential (Fig. 4f, Table 2). By evaluating the AUC of the two groups of PD patients, we observed that the AUC for the combined PTMs was greater than 0.8 (Fig. S6, Table S1 and Table S2). While in the subgroup 2–4 years the levels of AGEs allowed the greatest discrimination (Fig. S6a and Table S1), the levels of nY39 allowed the greatest discrimination in the group ≥ 10 years (Fig. S6b and Table S2). The combined analysis improved the AUC for both groups, and was greatest for the group ≥ 10 years (AUC = 0.877 ± 0.0451, 95% confidence interval 0.789–0.966, *p* < 0.0001) (Table S2).

**Table 2:** Diagnostic criteria of ROC curve (AUC) for the analysed PTMs between Parkinson’s disease patients and healthy individuals.

	AUC	95% Confidence interval	*p* value
AGE	0.807 ± 0.0479	0.713 to 0.900	<0.0001
SUMO	0.673 ± 0.0605	0.554 to 0.791	0.0082
pY125	0.754 ± 0.0540	0.648 to 0.860	<0.0001
nY39	0.733 ± 0.0523	0.631 to 0.836	0.0004
(A+p+n)/S	0.843 ± 0.0461	0.753 to 0.934	<0.0001

### The levels of PTMs correlate with disease severity and duration

**Figure 5:**
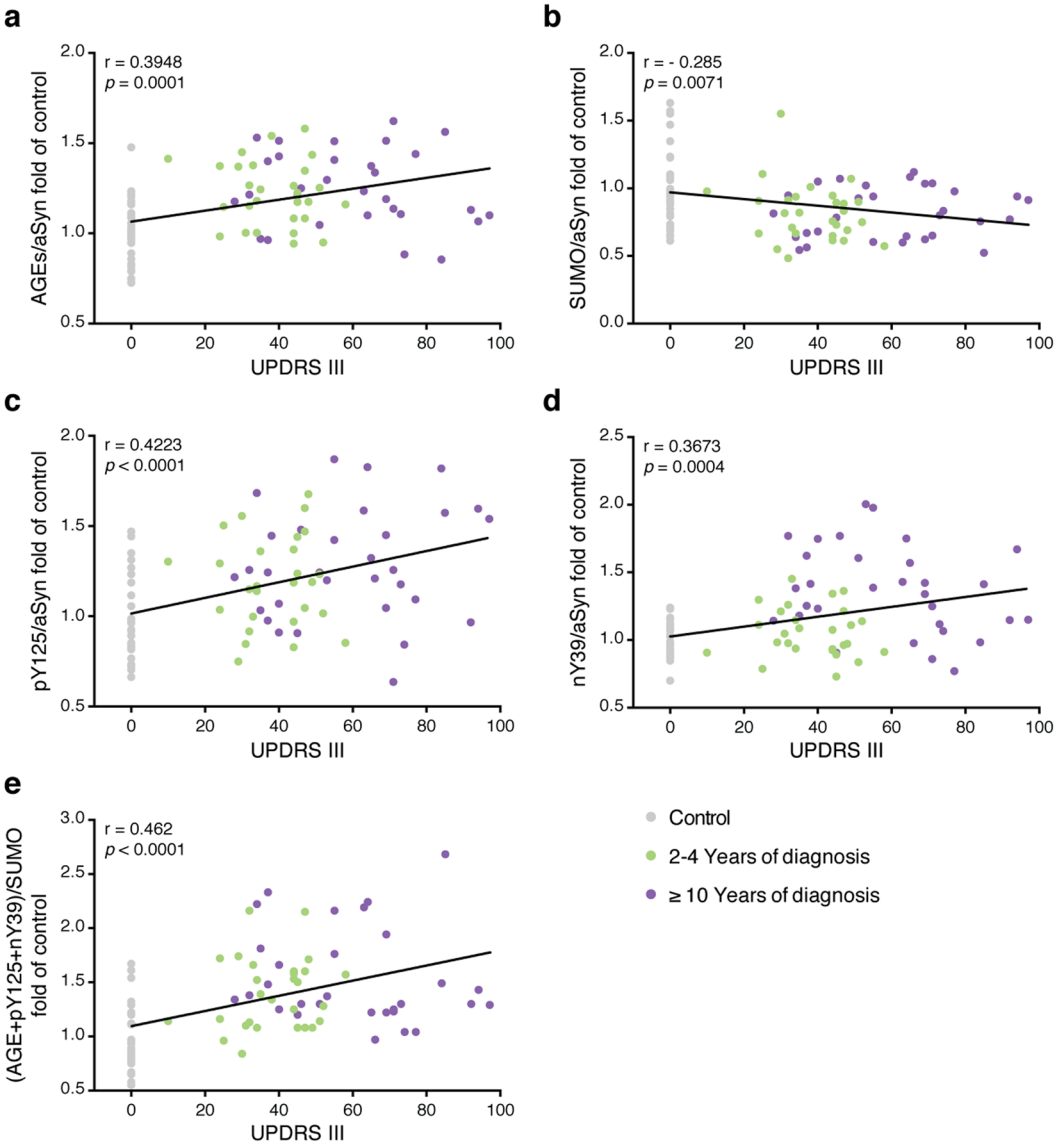
The levels of the PTMs correlate with MDS-UPDRS III. The correlation between the levels of glycation (**a**), SUMOylation (**b**), pY125 (**c**), nY39 (**d**) or of their combination (**e**) and UPDRS III scores was evaluated using linear regression analysis with Pearson’s correlation. The level of each individual PTM and corresponding UPDRS III score of healthy individuals (Control, grey), and patients between 2-4 years (green) or with ≥10 years (purple) is presented.

The correlation between the levels of single PTMs or their combination with PD severity (as determined by UPDRS or HY scores) was also examined. Impressively, all individual or the combination of PTMs correlated with the UPDRS motor score (UPDRS III) (*r* = 0.395, *p* < 0.0001, AGE; *r* = −0.285, *p* = 0.0072, SUMO; *r* = 0.422, *p* < 0.0001, pY125; *r* = 0.367, *p* = 0.0004, nY39; *r* = 0.462, *p* < 0.0001, (A + p + n)/S) (Fig. 5). All PTMs correlated with the sum of all UPDRS parts (Fig. S7). All PTMs except SUMOylation correlated with UPDRS I (Fig. S8), UPDRS II (Fig. S9), UPDRS IV scores (Fig. S10). All PTMs correlated with the HY score (Fig. S11).

**Figure 6:**
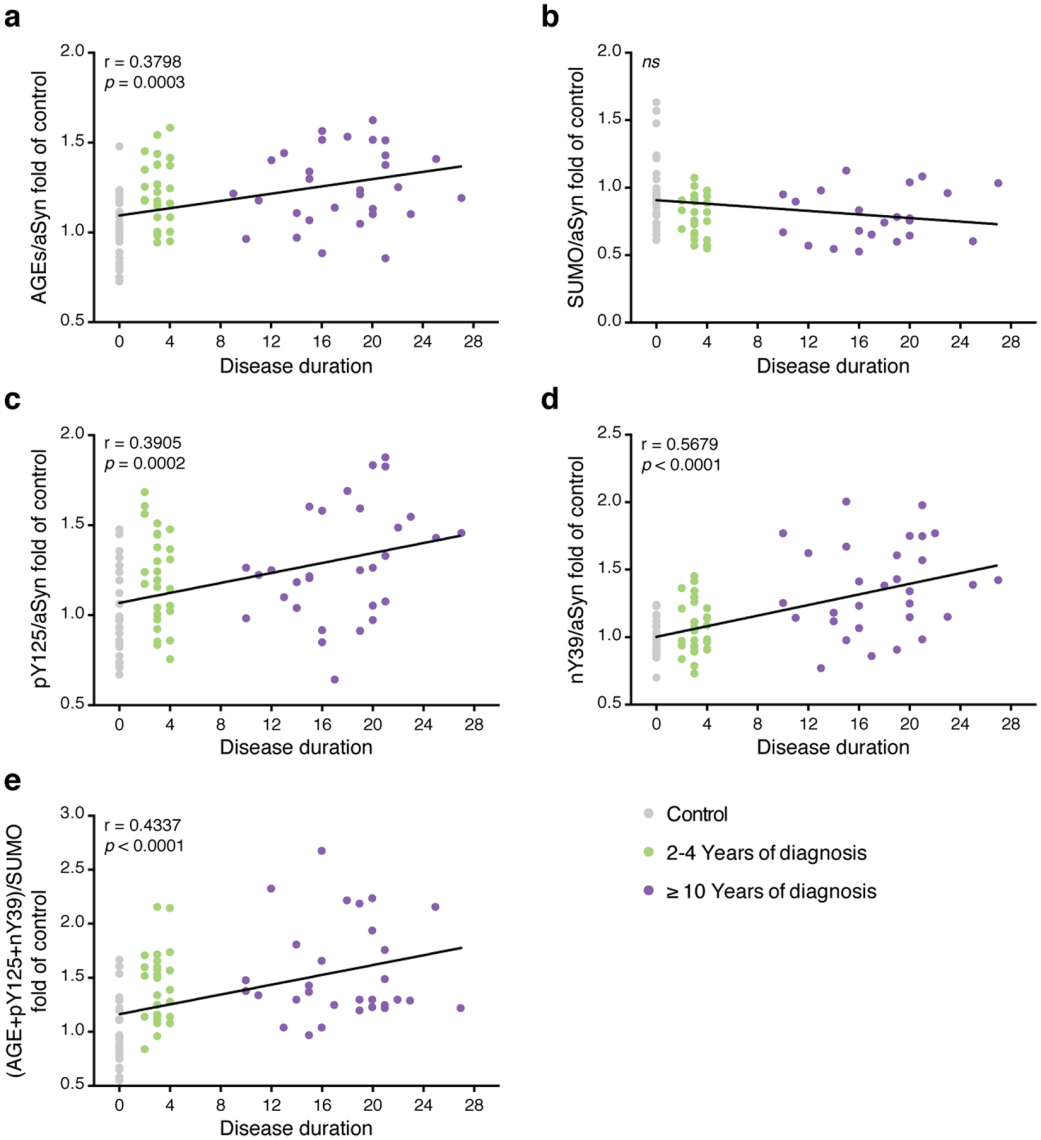
The levels of the PTMs correlate with disease duration. The correlation between the levels of glycation (**a**), SUMOylation (**b**), pY125 (**c**), nY39 (**d**) or of their combination (**e**) and disease duration was evaluated using linear regression analysis with Pearson’s correlation. The level of each individual PTM and corresponding disease duration of healthy individuals (Control, grey), and patients between 2-4 years (green) or with ≥10 years (purple) is presented.

We also evaluated the correlation between PTMs and disease duration. All PTMs, except SUMOylation, correlated with disease duration (*r* = 0.380, *p* = 0.0003, AGE; *r* = 0.3905, *p* = 0.0002, pY125; *r* = 0.5679, *p* < 0.0001, nY39; *r* = 0.4337, *p* < 0.0001, (A + p + n)/S) (Fig. 6).

Importantly, although the levodopa equivalent daily dose (LEDD) was significantly higher in the patients diagnosed for ≥10 years (Fig. S12a), LEDD did not correlate with the levels of PTMs (Fig. S12b–f).

## Discussion

The typical motor manifestations of PD appear when a significant percentage of nigral dopaminergic neurons have already been lost, and this is when clinical assessment enables the diagnosis, despite an unavoidable degree of uncertainty. There is hope that earlier diagnosis might enable earlier therapeutic interventions and, perhaps, the modulation of disease progression, both with existing as well as with future therapeutic options. Thus, the development of disease biomarkers is of great relevance. However, no clear objective diagnostic tool is currently available, despite tremendous efforts. Currently, among several promising ongoing studies, the value of measuring the levels of aSyn in the CSF of PD patients^37–40^, or the levels of phosphorylated S129 and oligomeric aSyn^41–43^ is being investigated. More recently, a panel of different peptides was also suggested as a putative biomarker of PD^44^. Notably, most reported candidates as biomarkers are proteins that are associated with PD and that are measured in peripheral tissues, reflecting central neuropathological alterations in PD. For example, the phosphorylation of aSyn on S129 is related to the aggregation and toxicity of aSyn^19,45^. Thus, we hypothesized that analyzing aSyn in easily accessible body fluids, where the protein is present, may report on characteristic modifications occurring in PD. aSyn is present in the CSF^46^ and also in the blood, including in erythrocytes^30^. However, the signature of aSyn PTMs has not been previously explored as putative biomarkers of PD.

Here, we used immunoassays to measure different PTMs in aSyn-enriched extracts from blood erythrocytes, easily accessible through a routine blood collection. We took advantage of the thermo-stability of aSyn and of haemoglobin depletion^35^, in order to facilitate the immunodetection of aSyn. Although this approach is not compatible with the study of the oligomerization state of aSyn, which is also likely to be of relevance, it provides complementary information that cannot be easily obtained in crude extracts under more native conditions. Thus, in future studies, it may also be valuable to conduct a parallel assessment of the oligomerization status of aSyn in patient-derived erythrocytes. Moreover, as previously suggested^47,48^, it may also be important to assess if putative aSyn tetramers or multimers are present at different levels in PD. Importantly, we found that a panel of selected PTMs is altered in PD patients, and discriminates healthy controls from diseased individuals. Importantly, the PTMs tested are not affected by the LEDD. We identified an increase in glycation, aSyn Y39 nitration and Y125 phosphorylation and a decrease in SUMO-1 levels in PD. Moreover, the combination of all PTMs improved the ability to discriminate between PD and healthy controls (AUC = 0.836 ± 0.0460). We also tested other known PTMs, however due to technical issues with the antibodies tested, no reproducible data was obtained and these were discarded from analysis. These included, for example, the widely-studied serine 129 (S129) phosphorylation, and aSyn acetylation. No detection was observed using the gold-standard antibody for S129 phosphorylation (Wako clone pSyn #64).

PTMs are known regulators of protein folding/structure, localization, and function and, therefore, play major roles in protein biology^49^. Here, we showed that the levels of glycation are increased in PD, possibly reflecting alterations also taking place in the brain^15,50,51^. Remarkably, we recently showed that glycation occurs in the brain, and that this PTM increases with aging (in animal models of the disease). Moreover, this modification is highly deleterious, potentiating neuronal loss and promoting motor impairment in a fly model of synucleinopathy^15^. Interestingly, we observed that the levels of glycation are also increased in the blood of PD patients. aSyn is also phosphorylated in tyrosine residues^52–55^ and pY125 was shown to prevent aSyn neurotoxicity and aggregation^55,56^. Surprisingly, we detected an increase in the levels of aSyn pY125 in the blood. As this modification occurs in PD^57,58^ and facilitates phosphorylation on S129, we hypothesize that the increase in pY125 in the blood from PD patients might reflect a connection with the pathogenicity of aSyn. Recent studies showed that SUMOylated aSyn is present in cell and animal models of PD^28,59^ and that SUMOylation increases aSyn solubility and reduces aggregation, both *in vitro* and *in vivo* ^28^. As we observed that the levels of SUMO-1 were reduced in PD-derived samples, this may increase the aggregation and toxicity of aSyn. Several studies reported the presence of nitrated aSyn in pathological conditions both *in vivo* and *in vitro*, and also in LBs^60,61^. In our study, we found increased levels of nY39 aSyn in samples from PD patients.

In several previous studies of biomarkers of PD, only moderate or no correlation with disease duration or severity was demonstrated^37,43,62–64^. Here, we also investigated the association of the levels of the PTMs with disease duration and severity, by correlating with the UPDRS and HY scores. We found that the levels of most PTMs correlate with parts of the UPDRS and HY scales. For example, the combination of the levels of the PTMs is well correlated with the motor UPDRS (*r* = 0.424, *p* < 0.0001). In our cohort, the patients diagnosed with PD for at least 2 years, have already an average UPDRS III score of 50 ± 19. Thus, it will be important to evaluate this correlation in *de novo* diagnosed PD patients. Nevertheless, as previously reported^41^, such patients could still be misdiagnosed or present low UPDRS scores, complicating diagnosis and treatment. Finally, we also observed an association between the levels of the PTMs with disease duration.

In contrast to a previous report on the total levels of aSyn in the CSF^37^, aging was not a confounding effect in the case of the levels of the PTMs. Taken together, our data suggest an association between the levels of the PTMs studied and disease duration and progression.

In future studies, we will employ more sensitive assays and increase the size of the cohorts, will include *de novo* diagnosed PD patients, and will perform a longitudinal study of the levels of PTMs to better understand the robustness of the assay. Quantitative mass-spectrometry-based techniques, would be ideal for this validation. In addition, it will be important to assess if the levels of these PTMs enable the discrimination between PD and other synucleinopathies or other neurodegenerative diseases.

In conclusion, our study identifies a panel of PTMs in the blood that is altered in PD patients and that may, therefore, constitute a novel biochemical marker for PD.

## Materials and Methods

### Patient selection

PD patients and controls were recruited from the Movement Disorders Outpatient Clinic at the Hospital de Santa Maria in Lisbon and consecutively invited to participate if presenting disease duration of 2-4 years or ≥10 years (age at first symptom). All patients signed an informed consent before any study related procedure. The study was approved by the Ethics Review Board of the Hospital de Santa Maria, Lisbon. All experiments were performed in accordance with the institution guidelines and regulations. Informed consent was obtained from all participants. PD patients were analyzed in the “on” period and where all under appropriate medication.

### Blood collection and processing

Blood samples were collected per participant by venipuncture into three EDTA-containing tubes. To isolate the erythrocytes, freshly drawn blood was centrifuged at 2000 g at 4 °C. Erythrocytes were washed 3 times with Phosphate Buffered Saline (PBS) (Gibco, Invitrogen, Spain), followed by 10 min centrifugation at 2000 g at 4 °C. Erythrocytes were stored at −80 °C until further use.

### Immunoblotting analysis

Samples (5 μg) were either loaded on pre-casted gels (4–15%, Bio-Rad, USA) according to standard procedures, or applied onto nitrocellulose membranes using a dot-blot system. In SDS-PAGE, prestained standard proteins (Thermo scientific, USA) were also loaded on the gel. Gels were either stained using coomassie brilliant blue (Bio-Rad, USA, 48 h) or transferred to nitrocellulose membrane (Bio-Rad, USA) using the Trans-Blot System (Bio-rad, USA) for immunoblotting analysis.

Membranes were blocked overnight at 4 °C in Tris-HCL buffer saline (TBS) (150 mM NaCl, 50 mM Tris pH 7.4) supplemented with 5% BSA. Membranes were then incubated overnight at 4 °C with different primary antibodies: mouse anti-Advanced Glycation and Products (AGEs) (KAL-KH001 Cosmo-Bio, USA, 1:500 dilution); rabbit anti-SUMO-1 (SUMO) (sc-9060 Santa Cruz Biotechnology, 1:1000 dilution); mouse anti-nitro-α/β-Synuclein (nY39-aSyn) (36-012, Upstate/Millipore, USA, 1:1000 dilution); rabbit anti-phosphorylated aSyn (pY125) (ab10789, Abcam, UK, 1:500 dilution); anti-ubiquitin (ab24686, Abcam, UK, 1:500 dilution); and mouse anti-aSyn (#610787, BD Transduction Laboratories, USA, 1:1000 dilution). All antibodies were diluted in 5% BSA in TBS. Membranes were incubated with the corresponding secondary antibodies prepared in TBS with 5% BSA for 2 h (AGEs, nY39-aSyn and aSyn with anti-mouse − 1:5000; SUMO-1 with anti-rabbit − 1:5000; PY125-aSyn with anti-rabbit − 1:1000). Detection procedures were performed using standard procedures for the ECL system (Millipore, USA) using appropriate exposure time in a Chemidoc system (Bio-Rad, XRS^+^).

### Measurement of PTM levels

After Western blot analysis of TE-HD fractions, densitometry of spots was performed using Image J^65^. The ratio between the levels of the selected PTM and those total aSyn was calculated. The levels of PTMs were then normalized to the average of the control group. Data is presented as PTM/aSyn fold of control.

### Statistical analyses

All statistical analyses were performed with Prism (Graphpad). For the case of statistical differences between healthy and diseased groups, we performed ordinary one-way ANOVA followed by Tukey’s multiple comparisons test. Linear regression and correlation analyses were performed to determine the relationships between the PTMs levels and age, disease duration or disease severity (MDS-UPDRS or HY). ROC curves analyses were used to calculate the relationship between the specificity and sensitivity for each disease group (2–4; ≥ 10; PD) versus healthy individuals, to evaluate the diagnostic and performance potential of the analytes. Values with *p* < 0.05 were considered significant.

## Electronic supplementary material

Supplementary Information
