# Supplementary material for: Posttranslational modifications of blood-derived alpha-synuclein as biochemical markers for Parkinson’s disease
Source: Sci Rep. 2017 Oct 20;7:13713. doi: 10.1038/s41598-017-14175-5 (PMC5651848; doi:10.1038/s41598-017-14175-5)
Supplement: Supplementary file 1 — Supplementary Information [file 41598_2017_14175_MOESM1_ESM.pdf]

TITLE

Posttranslational modifications of blood-derived alpha-synuclein as biochemical markers for Parkinson's disease

AUTHORS

Hugo Vicente Miranda*, Rafaela Cássio, Leonor Correia-Guedes, Marcos António Gomes, Ana Chegão, Elisa Miranda, Tiago Soares, Miguel Coelho, Mário Miguel Rosa, Joaquim J. Ferreira, and Tiago F. Outeiro*

SUPPLEMENTAL MATERIAL AND METHODS

Patient selection details

Groups were defined as 2-4 years to be able to recruit patients in early disease stages, but with more than 2 years from diagnosis in order to improve diagnostic accuracy. ≥ 10 years was defined to recruit patients with long disease duration. Controls were spouses or carers of patients with no known chronic diseases, no signs of parkinsonism and no family history of neurodegenerative disorders. Demographic and clinical data collection and patients' examination was performed by a neurologist specialized in Parkinson's disease. MDS-UPDRS and HY scales were applied to all Parkinson's disease patients for rating symptoms, motor signs and disease progression. Blood samples were collected, aSyn was partially purified from erythrocytes, and selected PTMs were measured using immunoblotting. The levodopa-equivalent daily dose (LEDD) was determined according to the latest guidelines [1].

Compliance with Ethical Standards

All procedures performed in studies involving human participants were in accordance with the ethical standards of the institutional and/or national research committee and with the 1964 Helsinki declaration and its later amendments or comparable ethical standards.

Informed Consent

Informed consent was obtained from all individual participants included in the study.

Erythrocyte lysis

Cells were lysed using NP-40 lysis buffer supplemented with a mixture of proteases and phosphatases inhibitors (Complete-Mini, EDTA-free and PhosStop, Roche) and sonicated (Soniprep 150) three times for 10 s at 10 µm amplitude. Cells were further lysed by ultra-freezing in liquid nitrogen. Final extracts were centrifuged at 20200 g, 4° C for 10 min and the supernatant collected and readily used, or stored at -80° C until further use. Erythrocyte samples were randomized for analysis, with both control and Parkinson's disease samples being assayed simultaneously in the same apparatus.

aSyn thermoenrichment and purification

To detect aSyn modifications, we performed a partial purification and enrichment of aSyn from erythrocytes lysates (at least n=4). First, we adapted a method that we previously described [2]. Briefly, we incubated 150 mg of protein at 90° C for 15 min, centrifuged at 20200 g, 4° C for 30 min, and collected the supernatant. Afterwards, since haemoglobin is the major protein present in erythrocytes, we depleted the remaining haemoglobin using the Hemovoid kit (Biotech Support Group) according to manufacturer procedures [3]. The eluted fractions were collected and concentrated in a speedvac concentrator (Thermo Scientific). Samples were readily used or stored at -80° C.

Protein measurement

Total protein was measured using Thermo Scientific™ Pierce™ BCA Protein Assay that uses a detergent-compatible formulation based on bicinchoninic acid (BCA). Protein concentrations were determined with reference to BSA according to standard procedures.

Dot blot analysis

Eluted fractions (5 μg) were applied in nitrocellulose membrane (0.22 μm pore; GE Water & Process Technologies, Fairfield, USA), previously incubated with PBS, using a dot-blot system (Scie-Plas, UK) by vacuum. Immunobloting was performed as previously described. This procedure was performed at least 4 times.

SUPPLEMENTAL FIGURES

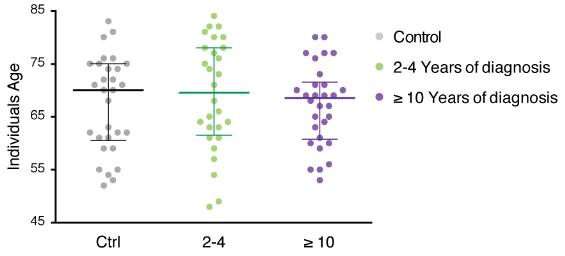

Fig. S1. Mean age of cohort. The age of the healthy individuals (Control, grey) and patients with disease duration between 2-4 years (green) or ≥ 10 years (purple) is presented.

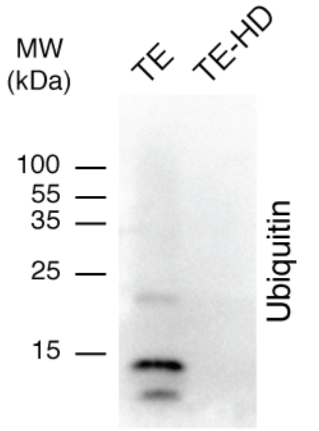

Fig. S2. Ubiquitin is not detected in thermo-enriched-haemoglobin-depleted samples. Western blot analysis of an SDS-PAGE separation of 5 µg of TE and TE-HD probed with an antibody against ubiquitin.

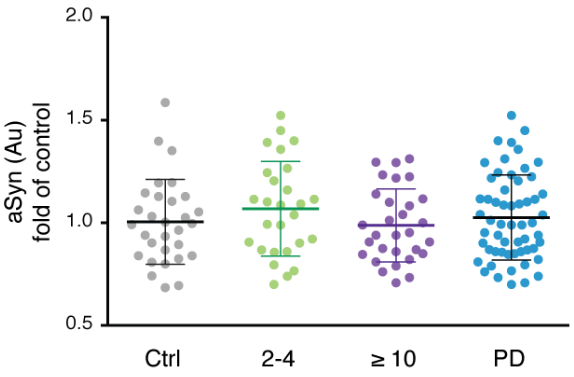

Fig. S3. Mean levels of aSyn in erythrocytes of participating subjects. The mean levels of aSyn from healthy individuals (Control, grey), patients with disease duration between 2-4 years (green) or ≥ 10 years (purple), and all PD patients (blue) is presented.

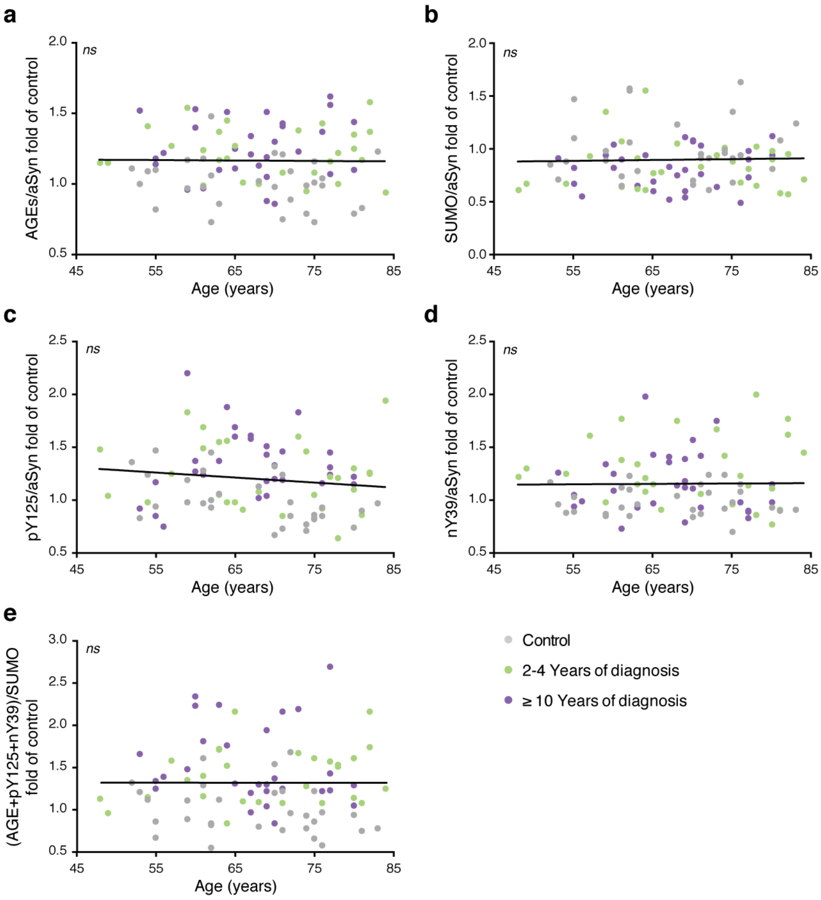

Fig. S4. The levels of the PTMs are not altered with aging. The correlation between the levels of glycation **(a)**, SUMOylation **(b)**, pY125 **(c)**, nY39 **(d)** or of their combination **(e)** and individuals age was evaluated using linear regression analysis with Pearson's correlation. The level of each individual PTM and corresponding age of healthy individuals (Control, grey), and patients between 2-4 years (green) or ≥ 10 years (purple) is presented.

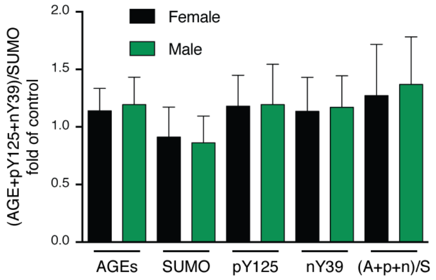

Fig. S5. The levels of the PTMs are similar between genders. The mean levels of all PTM and their combination of all participants according to their gender is presented.

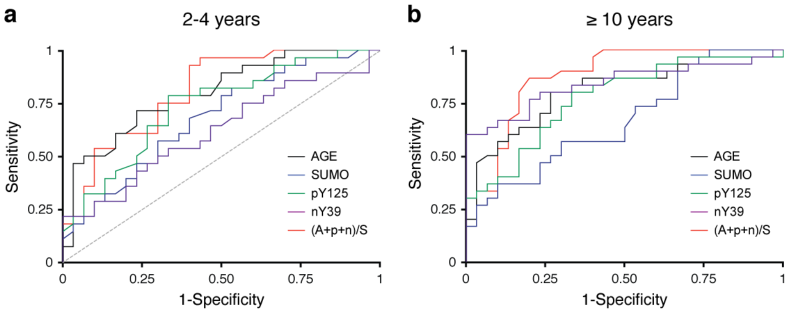

Fig. S6. The levels of the PTMs are specifically altered in PD patients. ROC curve to evaluate the utility of the PTMs in discriminating healthy controls from patients with PD (2-4 years) **(a)** or PD (≥ 10 years) **(b)**. Glycation (black), SUMO (blue), pY125 (green), nY39 (purple) and PTMs combination (red) curves are presented.

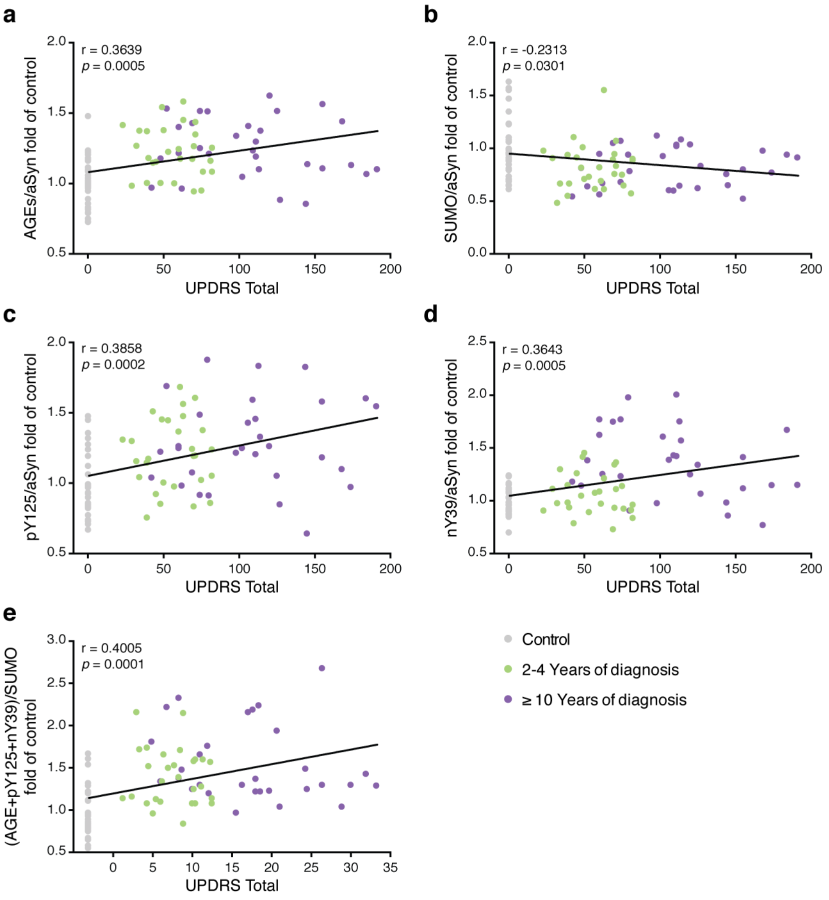

Fig. S7. The levels of the PTMs correlate with MDS-UPDRS Total scores. The correlation between the levels of glycation **(a)**, SUMOylation **(b)**, pY125 **(c)**, nY39 **(d)** or of their combination **(e)** and UPDRS total scores was evaluated using linear regression analysis with Pearson's correlation. The level of each individual PTM and corresponding UPDRS III score of healthy individuals (Control, grey), and patients between 2-4 years (green) or ≥ 10 years (purple) is presented.

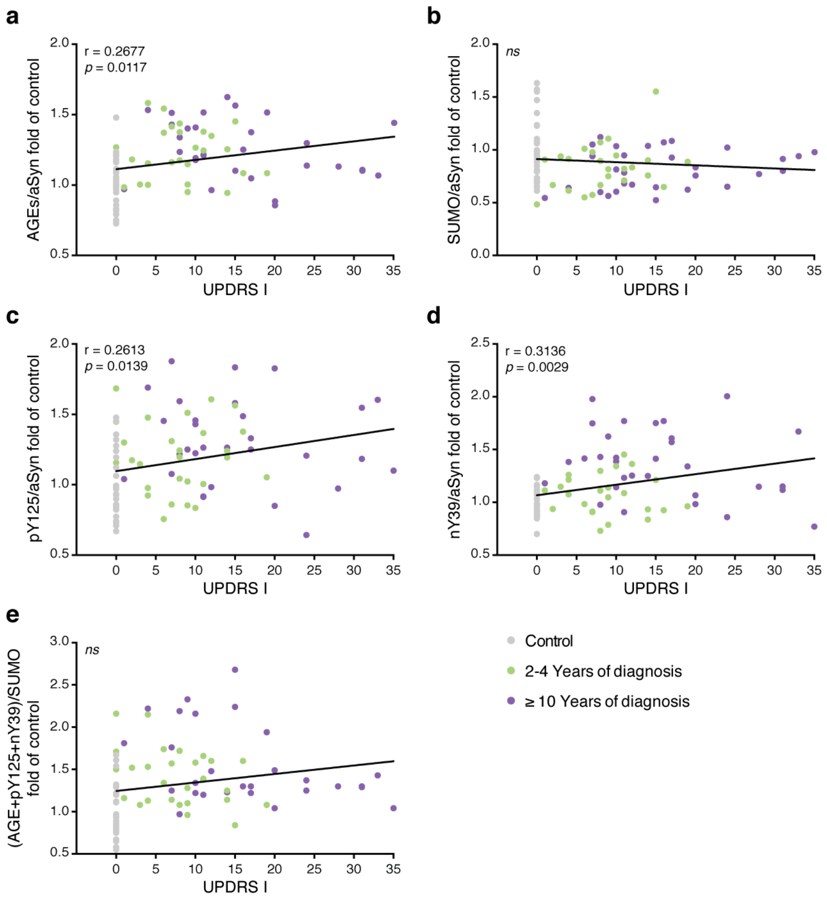

Fig. S8. The levels of the PTMs correlate with MDS-UPDRS I. The correlation between the levels of glycation **(a)**, SUMOylation **(b)**, pY125 **(c)**, nY39 **(d)** or of their combination **(e)** and UPDRS I scores was evaluated using linear regression analysis with Pearson's correlation. The level of each individual PTM and corresponding UPDRS III score of healthy individuals (Control, grey), and patients between 2-4 years (green) or ≥ 10 years (purple) is presented.

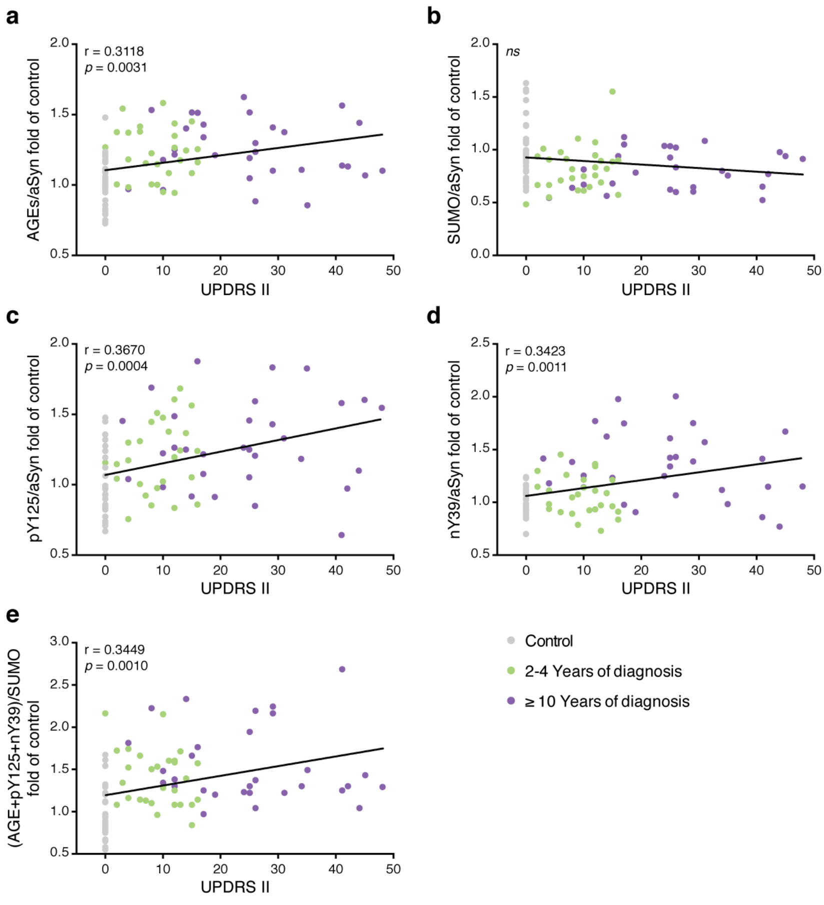

Fig. S9. The levels of the PTMs correlate with MDS-UPDRS II. The correlation between the levels of glycation **(a)**, SUMOylation **(b)**, pY125 **(c)**, nY39 **(d)** or of their combination **(e)** and UPDRS II scores was evaluated using linear regression analysis with Pearson's correlation. The level of each individual PTM and corresponding UPDRS III score of healthy individuals (Control, grey), and patients between 2-4 years (green) or ≥ 10 years (purple) is presented.

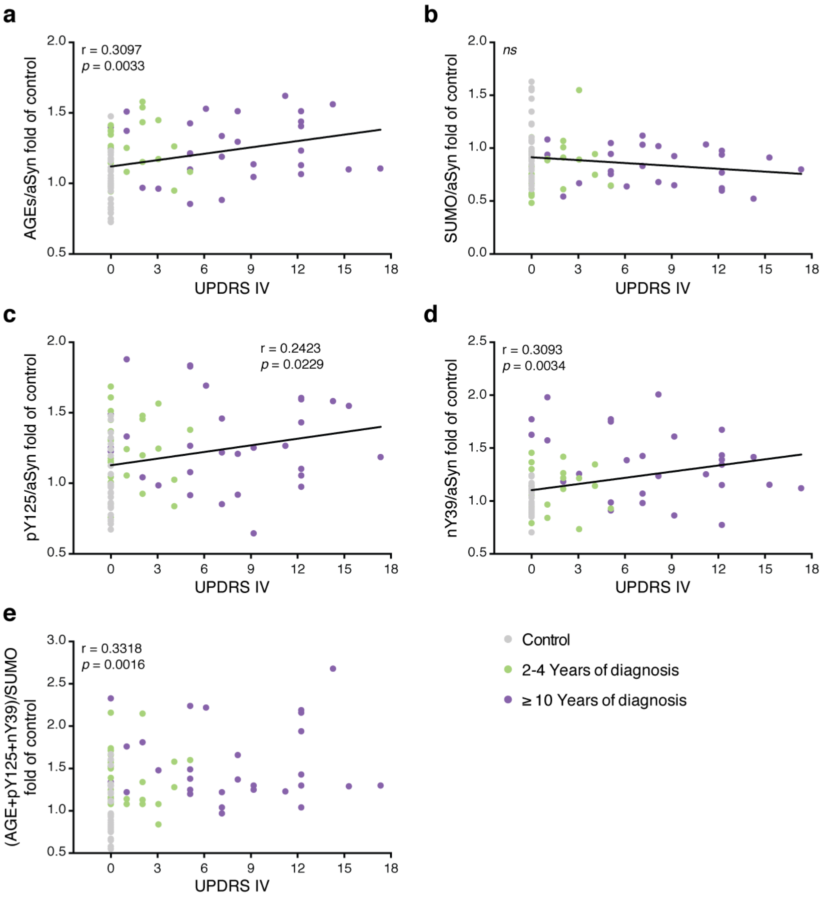

Fig. S10. The levels of the PTMs correlate with MDS-UPDRS IV. The correlation between the levels of glycation **(a)**, SUMOylation **(b)**, pY125 **(c)**, nY39 **(d)** or of their combination **(e)** and UPDRS IV scores was evaluated using linear regression analysis with Pearson's correlation. The level of each individual PTM and corresponding UPDRS III score of healthy individuals (Control, grey), and patients between 2-4 years (green) or ≥ 10 years (purple) is presented.

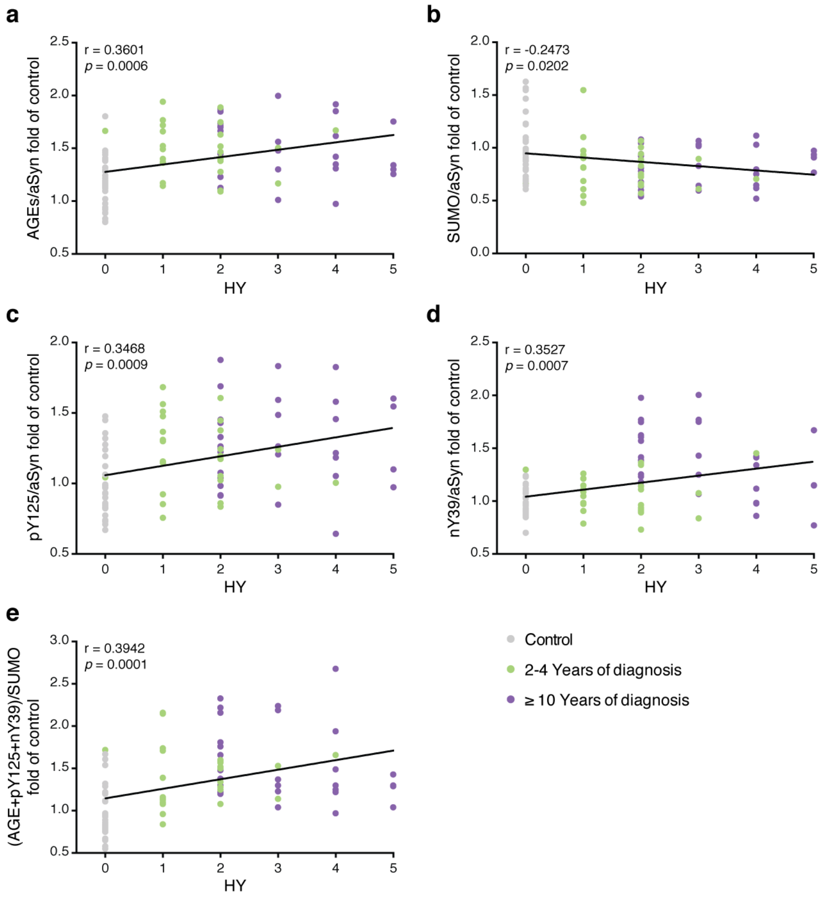

Fig. S11. The levels of the PTMs correlate with H&Y. The correlation between the levels of glycation **(a)**, SUMOylation **(b)**, pY125 **(c)**, nY39 **(d)** or of their combination **(e)** and H&Y scores was evaluated using linear regression analysis with Pearson's correlation. The level of each individual PTM and corresponding UPDRS III score of healthy individuals (Control, grey), and patients between 2-4 years (green) or ≥ 10 years (purple) is presented.

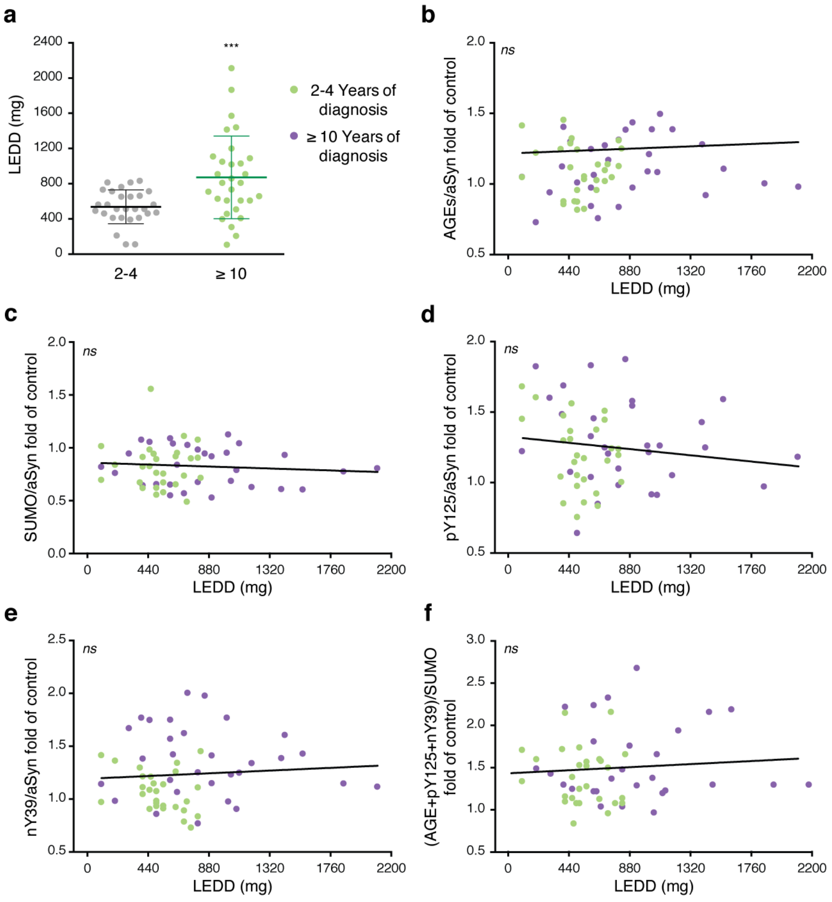

Fig. S12. The levels of the PTMs do not correlate with levodopa equivalent daily dose (LEDD). (a) The mean levels of LEDD from patients with disease duration between 2-4 years (green) or ≥ 10 years (purple) is presented. The correlation between the levels of glycation **(b)**, SUMOylation **(c)**, pY125 **(d)**, nY39 **(e)** or of their combination **(f)** and LEDD was evaluated using linear regression analysis with Pearson's correlation. The level of each individual PTM and corresponding LEDD of patients between 2-4 years (green) and ≥ 10 years (purple) is presented.

SUPPLEMENTAL TABLES

Table S1. Diagnostic criteria of ROC curve (AUC) for analysed PTMs between patients with disease duration of 2-4 years and healthy individuals.

	AUC	95 % Confidence interval	*p* value
AGE	0.799 ± 0.0579	0.685 to 0.912	<0.0001
SUMO	0.678 ± 0.0710	0.539 to 0.817	0.0200
pY125	0.734 ± 0.0656	0.605 to 0.862	0.0022
nY39	0.619 ± 0.0745	0.472 to 0.765	ns
(A+p+n)/S	0.807 ± 0.0565	0.696 to 0.917	< 0.0001

Table S2. Diagnostic criteria of ROC curve (AUC) for analysed PTMs between patients with disease duration of ≥10 years and healthy individuals.

	AUC	95% Confidence interval	*p* value
AGE	0.814 ± 0.0551	0.706 to 0.922	<0.0001
SUMO	0.668 ± 0.0670	0.531 to 0.805	0.0256
pY125	0.773 ± 0.0605	0.654 to 0.891	0.0003
nY39	0.840 ± 0.0539	0.734 to 0.946	<0.0001
(A+p+n)/S	0.877 ± 0.0451	0.789 to 0.966	<0.0001

SUPPLEMENTAL REFERENCES

1 Tomlinson, C. L. *et al.* Systematic review of levodopa dose equivalency reporting in Parkinson's disease. *Mov Disord* **25**, 2649-2653, doi:10.1002/mds.23429 (2010).

2 Vicente Miranda, H. *et al.* Heat-mediated enrichment of alpha-synuclein from cells and tissue for assessing post-translational modifications. *J Neurochem* **126**, 673-684, doi:10.1111/jnc.12251 (2013).

3 Walpurgis, K. *et al.* Validated hemoglobin-depletion approach for red blood cell lysate proteome analysis by means of 2D PAGE and Orbitrap MS. *Electrophoresis* **33**, 2537-2545, doi:10.1002/elps.201200151 (2012).
